# Mono- and combination drug therapies in hospitalized patients with bipolar depression. Data from the European drug surveillance program AMSP

**DOI:** 10.1186/1471-244X-12-153

**Published:** 2012-09-21

**Authors:** Anne Haeberle, Waldemar Greil, Stefan Russmann, Renate Grohmann

**Affiliations:** 1 Department of Psychiatry, Ludwig Maximilian University, Munich, Germany, Nussbaumstr. 7, Munich, 80336, Germany; 2 Sanatorium Kilchberg, Alte Landstrasse 70, Kilchberg-Zurich, 8802, Switzerland; 3 Department of Clinical Pharmacology and Toxicology, University Hospital Zurich, Raemistrasse 100, Zurich, 8091, Switzerland

**Keywords:** AMSP, Bipolar depression, Bipolar disorder, Combination therapy, Drug surveillance, Guidelines, Pharmacotherapy, Prescription, Psychotropic drugs, Antidepressants

## Abstract

**Background:**

For the pharmacological treatment of bipolar depression several guidelines exist. It is largely unknown, to what extent the prescriptions in daily clinical routine correspond to these evidence based recommendations and which combinations of psychotropic drugs are frequently used.

**Methods:**

The prescriptions of psychotropic drugs were investigated of all in-patients with bipolar depression (n = 2246; time period 1994–2009) from hospitals participating in the drug surveillance program AMSP. For the drug use in 2010, 221 cases were analysed additionally.

**Results:**

From 1994 to 2009, 85% of all patients received more than one class of psychotropic substances: 74% received antidepressants in combination therapy, 55% antipsychotics, 48% anticonvulsants and 33% lithium. When given in combination, lithium is the most often prescribed substance for bipolar depression (33%), followed by valproic acid (23%), mirtazapine and venlafaxine (16% each), quetiapine (15%), lamotrigine (14%) and olanzapine (13%). Both, lithium and valproic acid are often combined with selective serotonin reuptake inhibitors (SSRI), but also with mirtazapine und venlafaxine. Combinations of more than one antidepressant occur quite often, whereby combinations with bupropion, paroxetine, fluoxetine or fluvoxamine are very rare. In 2010, quetiapine (alone and combined) was the most frequently prescribed drug (39%); aripiprazole was administered in 10%.

**Conclusion:**

Combinations of antidepressants (SSRI, mirtazapine, venlafaxine) with mood stabilizers (lithium, valproic acid, lamotrigine) and / or atypical antipsychotics (quetiapine, olanzapine) are common. Of most of those combinations the efficacy has not been studied. The use of aripiprazole and the concomitant use of two or three antidepressants contrast the guidelines.

## Background

For the treatment of bipolar depression a variety of partly controversial options exist. Several up to date guidelines provide clinicians with a framework of evidence based pharmacological treatments of bipolar depression [1-6].

The present study shows the prescriptions of psychopharmacological substances for bipolar depression in daily clinical routine. The data from a large European multicenter study (AMSP) [7,8] allow a direct comparison between clinical routine and recommendations of the guidelines.

Previous analysis of these data have shown several important prescribing trends in the treatment of bipolar depression from 1994 until 2009: Antidepressants are prescribed in almost 80% of all in-patients, thus being by far the most important class of psychotropic drugs prescribed for bipolar depression; antipsychotics in about 60% with increasing trend, especially for quetiapine; anticonvulsants in about 50%, mostly valproic acid and lamotrigine and lithium in about 35% with a decreasing trend. Furthermore, a pronounced increase of polypharmacy was found [9].

For the first time, the present article investigates specifically the most frequently administered combinations of psychotropic substances and focuses on the use of single antidepressant drugs over the time of 15 years in hospitalized patients with bipolar depression.

## Method

### Patients

All in-patients with a diagnosis of bipolar depression hospitalized in the participating hospitals of the AMSP project were selected (n = 2246; time period 1994–2009). For the years 2001 – 2009 patients with the diagnoses F 31.3 – 31.5 according to ICD 10 were selected, for the years 1994 – 2000 patients with the corresponding diagnosis in ICD 9 (manic-depressive psychosis, circular type, currently depressed) were included. A detailed description of the patient population can be found in Greil et al. 2012 [9]. For the present analysis 15 patients with missing personal data not included in the previous analysis were added to the population. For the special calculation of drug use in 2010 additional 221 cases were analysed.

### Data collection

The present data were taken from the large data pool of the AMSP program (Arzneimittelsicherheit in der Psychiatrie) [7]. This drug safety project was started in 1993 by the Psychiatric University Hospital Munich and serves to continuously record prescriptions of psychotropic drugs and their adverse side effects of in-patients of various hospitals in Germany, Switzerland and Austria (and temporarily also Hungary and Belgium). In 2009, 51 hospitals participated in the project. The data on prescription rates were gathered twice a year as follows: At two index dates per year each participating hospital / ward recorded for each patient hospitalized at that day age, sex, psychiatric diagnosis (ICD 9 and 10, respectively) and daily dosage of all drugs (psychotropic and non-psychotropic). These data were subsequently sent to the Psychiatric University Hospital Munich, where they were collected in an overall database.

### Data analysis

To statistically analyse the rates of psychopharmacological prescriptions, the percentage of patients per year was calculated, which received a particular agent. The number of patients receiving a particular agent in one year was divided by the total number of patients in this year = prescription rate, i.e. percentage of patients receiving the agent per year. Although the data were collected twice per calendar year, an average prescription rate was calculated using the data of one calendar year. Hence, we phrase “% of patients” referring to virtual single patients. For the analyses, the period 1994–2009 was segmented into four equal time periods due to low number of patients with single drugs.

The ethics committee of the Ludwig Maximilian University of Munich, the location of the AMSP main data center, had approved the analysis of the AMSP data with a waver of authorization. The permission to use the special data set of bipolar depression was given by the publication commission consisting of the presidents of the AMSP associations in Germany, Austria and Switzerland.

## Results

### Frequency of prescriptions of drug classes in combination therapy

Table 1 shows that within the period from 1994 to 2009, 85% of all hospitalized patients treated for bipolar depression received more than one class of psychotropic substances: 74% of all patients received antidepressants, 55% antipsychotics, 48% anticonvulsants and 33% lithium in combination therapy, i.e. in combination with other drugs of these four drug classes.

**Table 1:** Frequency of prescriptions in mono- and combination therapy for classes of psychotropic substances

		**1994 - 1997**		**1998 - 2001**		**2002 - 2005**		**2006 - 2009**		**Overall**	
		**N**	**%**	**N**	**%**	**N**	**%**	**N**	**%**	**N**	**%**
Number of patients		248		454		711		832		2246	
Number of patients monotherapy		53	**21.4**	99	**21.8**	85	**12.0**	97	**11.7**	334	**14.9**
Number of patients combination therapy		195	**78.6**	355	**78.2**	626	**88.0**	735	**88.3**	1912	**85.1**
Antidepressants	monotherapy	33	**13.3**	54	**11.9**	41	**5.8**	43	**5.2**	171	**7.6**
	combination therapy	172	**69.4**	317	**69.8**	531	**74.7**	634	**76.2**	1655	**73.7**
Antipsychotics	monotherapy	8	**3.2**	16	**3.5**	16	**2.3**	30	**3.6**	70	**3.1**
	combination therapy	97	**39.1**	194	**42.7**	418	**58.8**	534	**64.2**	1244	**55.4**
Anticonvulsants	monotherapy	6	**2.4**	16	**3.5**	15	**2.1**	14	**1.7**	51	**2.3**
	combination therapy	65	**26.2**	177	**39.0**	402	**56.5**	425	**51.1**	1069	**47.6**
Lithium	monotherapy	6	**2.4**	13	**2.9**	13	**1.8**	10	**1.2**	42	**1.9**
	combination therapy	105	**42.3**	152	**33.5**	204	**28.7**	275	**33.1**	736	**32.8**

Monotherapy had a low prevalence (about 15% of the patients) and shows a decreasing trend. A remarkable decrease is seen for antidepressant monotherapy, from 13% to 5% in the time period from 1994 to 1997 as compared to the last period from 2006 to 2009. Hereby, monotherapy is defined as the use of either antidepressants or antipsychotics or anticonvulsants or lithium, but additional use of hypnotics and tranquilizers or the use of more than one drug *within* the respective class being allowed (see also [9]).

### Psychotropic substances in mono- and combination therapy

Table 2 shows the single psychotropic substances prescribed most frequently in mono- as well as in combination therapy. When given in combination, lithium is the most often prescribed substance for bipolar depression (33%), followed by valproic acid (23%), mirtazapine and venlafaxine (16% each), quetiapine (15%), lamotrigine (14%) and olanzapine (13%). The group of SSRI is frequently included in combination therapy (26% of the patients), especially escitalopram (9%) and citalopram (8%). Note that paroxetine and fluoxetine are prescribed rarely and fluvoxamine as well as bupropion are given only exceptionally. Interestingly, quetiapine and lamotrigine were often administered as combination therapy, but very rarely as monotherapy (0.6% and 0.3%, respectively).

**Table 2:** Frequency of prescriptions of single psychotropic substances

**%**	**1994 - 1997**	**1998 – 2001**	**2002 – 2005**	**2006 - 2009**	**Overall**
N	248	454	711	832	2246
Lithium	2.4 + **42.3**	2.9 + **33.5**	1.8 + **28.7**	1.2 + **33.1**	1.9 + **32.8**
SSRI overall	2.4 + **15.3**	3.5 + **22.4**	2.4 + **29**	2.0 + **28.6**	2.5 + **26.0**
Valproic acid	0.4 + **4.8**	1.8 + **19.8**	1.5 + **28.4**	1.3 + **24.4**	1.4 + **22.8**
Mirtazapine	**3.2**	2.6 + **12.1**	2.1 + **20.7**	2.0 + **18.8**	2.0 + **16.3**
Venlafaxine	2.0 + **4.4**	1.1 + **10.4**	1.3 + **21.5**	1.1 + **18.3**	1.2 + **16.2**
Quetiapine		**0.7**	0.3 + **12.7**	1.4 + **28.1**	0.6 + **14.6**
Lamotrigine		0.7 + **2.9**	0.1 + **17.7**	0.4 + **22.2**	0.3 + **14.4**
Olanzapine	**0.4**	0.9 + **9.5**	0.7 + **18**	1.1 + **13.6**	0.8 + **12.7**
Carbamazepine	2.0 + **21**	1.8 + **18.7**	0.4 + **10.4**	0.2 + **5.5**	0.8 + **11.4**
Escitalopram			0.7 + **10.1**	1.1 + **14.4**	0.6 + **8.5**
Citalopram	0.8 + **4.8**	1.3 + **8.1**	0.8 + **9.8**	0.2 + **7**	0.7 + **7.9**
Risperidone	**1.6**	0.2 + **5.9**	0.1 + **7.3**	0.2 + **7.6**	0.2 + **6.5**
Sertraline	**0.4**	0.9 + **6.4**	0.4 + **5.1**	0.5 + **4.0**	0.5 + **4.4**
Paroxetine	0.8 + **8.5**	1.3 + **5.3**	0.1 + **2.5**	**2.4**	0.4 + **3.7**
Perazine	0.8 + **5.6**	0.4 + **5.3**	**3.1**	**1.3**	0.2 + **3.2**
Reboxetine		1.1 + **2.9**	0.1 + **4.2**	0.2 + **3.0**	0.4 + **3.0**
Haloperidol	0.8 + **11.3**	0.4 + **4.0**	**1.5**	0.7 + **1.3**	0.4 + **3.0**
Duloxetine			0.1 + **1.5**	0.1 + **6.6**	0.1 + **2.9**
Melperone	**2.0**	0.2 + **3.3**	0.4 + **3.7**	**2.4**	0.2 + **2.9**
Promethazine	0.8 + **5.6**	0.2 + **3.1**	0.1 + **1.4**	0.5 + **1.7**	0.4 + **2.3**
Aripiprazole			**1.5**	0.1 + **4.7**	**2.0**
Clozapine	**3.2**	0.2 + **2.2**	0.3 + **1.7**	0.1 + **1.8**	0.2 + **2.0**
Fluoxetine	0.8 + **1.6**	**2.6**	0.4 + **1.5**	0.2 + **0.8**	0.3 + **1.5**

Table 2: Monotherapy (small numbers) and combination therapy (fat numbers) in percent (%) of all patients per time period. The substances are listed according to their frequency of prescription, with the most often prescribed substance listed first. Substances with overall prescription rates below 1.5% are not presented. Year of licensing in the respective countries: lamotrigine: epilepsy 1994, long term bipolar depression 2003; olanzapine: schizophrenia 1996, long term mania 2003; quetiapine: schizophrenia 2000, mania 2004, bipolar depression 2009.

The analysis of the prescription data from 2010 (patients with bipolar depression, n = 221) shows the following results for the most often used single drugs: Quetiapine (alone and in combination) is prescribed in 38.9% of the patients, followed by valproic acid (33.5%), lithium (26.7%), escitalopram (19.5%), lamotrigine (18.6%), venlafaxine (17.7%) and mirtazapine (14.5%). Interestingly, aripiprazole (10.0%) is prescribed more often than olanzapine (9.1%). The group of SSRI is prescribed in 30.9%.

### Frequent combinations of psychotropic substances

Table 3 a and b gives the most frequent combinations of at least two or three psychotropic substances in the time period from 1994 to 2009. The results show, that frequent combinations include lithium, SSRI, mirtazapine, venlafaxine, valproic acid, lamotrigine, quetiapine and olanzapine. Both, lithium and valproic acid are often combined with SSRI, but also with mirtazapine and venlafaxine. Particular combinations with risperidone are not frequent (< 3%) and hence not included in the table. Combinations with bupropion, fluoxetine, fluvoxamine or paroxetine are very rare and therefore not presented.

**Table 3:** a and b **Most frequent combinations of at least two and three substances**

**a.**			
Lithium	SSRI	**9.2%**	
Valproic acid	SSRI	**7.2**	
Lithium	Mirtazapine	**5.8**	
Lithium	Venlafaxine	**5.7**	
Quetiapine	SSRI	**5.2**	
Quetiapine	Lithium	**5.1**	
Lamotrigine	SSRI	**4.9**	
Valproic acid	Venlafaxine	**4.6**	
Valproic acid	Mirtazapine	**4.5**	
Valproic acid	Lithium	**4.1**	
Quetiapine	Lamotrigine	**3.9**	
SSRI	Mirtazapine	**3.8**	
Olanzapine	Lithium	**3.7**	
Lamotrigine	Venlafaxine	**3.7**	
Olanzapine	SSRI	**3.6**	
Quetiapine	Valproic acid	**3.4**	
Mirtazapine	Venlafaxine	**3.4**	
Lamotrigine	Lithium	**3.3**	
Valproic acid	Olanzapine	**3.2**	
Lamotrigine	Mirtazapine	**3.2**	
**b.**			
SSRI	Quetiapine	Lithium	**1.7%**
SSRI	Quetiapine	Lamotrigine	**1.3**
SSRI	Mirtazapine	Lithium	**1.2**
SSRI	Mirtazapine	Valproic acid	**1.2**
SSRI	Mirtazapine	Venlafaxine	**1.2**
Lithium	Mirtazapine	Venlafaxine	**1.2**
SSRI	Olanzapine	Lithium	**1.1**
Lithium	Quetiapine	Venlafaxine	**1.1**
Lithium	Quetiapine	Lamotrigine	**1.1**

### Frequent combinations of classes of substances

Table 4 shows a summary of the most frequent combinations of classes of psychotropic drugs in the treatment of bipolar depression. Most combinations include antidepressants, mainly SSRI. Interestingly, also combinations of two and more antidepressant drugs occur, e.g. SSRI plus mirtazapine plus venlafaxine.

**Table 4:** Most frequent combinations of classes of psychotropic drugs

Lithium	Antidepressants (SSRI, Mir, Ven)	atypical Neuroleptics (Quet, Ola, Ri)	**4%**
Lithium	Antidepressants (SSRI, Mir, Ven)	Antidepressants (SSRI, Mir, Ven)	**3%**
Lithium	atypical Neuroleptics (Quet, Ola, Ri)	Anticonvulsives (Val, Lam)	**1%**
SSRI	atypical Neuroleptics (Quet, Ola, Ri)	Anticonvulsives (Val, Lam)	**3%**
SSRI	Mirtazapine	Anticonvulsives (Val, Lam)	**2%**
SSRI	Mirtazapine	Venlafaxine	**1%**

## Discussion

The present study focuses on the psychopharmacological combination treatment of bipolar depression in hospitalized patients in the time period 1994–2009. It shows that lithium given in about one third of the patients is the most frequently administered single substance given at all and concomitantly with other psychotropic drugs. This is also found for the time period 2006–2009, even though the trend to prescribe lithium has decreased over the last 15 years [9]. The data, however, do not allow a specification whether lithium is given due to its antidepressant property, as an augmentation strategy or for preventive purpose.

Clinical studies on the efficacy of lithium show a modest antidepressant effect of lithium in bipolar depression, at best [10-13]. Nevertheless, several guidelines include lithium as a fist-line treatment option alone [4,6] or in combination with other substances, e.g. lamotrigine [2]. The combinations of lithium with other psychotropic drugs found in the present data only include substances that do not interfere with the pharmacokinetics of lithium. Only the risk of serotonergic syndrome may be increased in combinations of lithium with SSRI and / or venlafaxine.

The data also emphasize, that antidepressants are the most frequently prescribed class of drugs given in combination, although the use of antidepressants in bipolar depression is controversial. Since 2002, in US guidelines it is generally recommended to avoid antidepressants in bipolar depression [14]. Especially for mirtazapine and venlafaxine, both found in the present study to be combined often with lithium and valproic acid respectively, there is no data that supports the efficacy of these combinations.

In 2002, international [15] and US guidelines [14] proposed the combination of a non-tricyclic antidepressant (SSRI or bupropion) with mood stabilizer (lithium or lamotrigine) as a treatment option. In accordance with these recommendations and with modern guidelines, that in bipolar depression antidepressants should be prescribed in combination with mood stabilizing and antimanic drugs only we observed that antidepressants are combined mainly with lithium, valproic acid, quetiapine and lamotrigine. However, the evidence based combination recommended by the guidelines, olanzapine plus fluoxetine (OFC), was found to be prescribed only in very few patients (cf. [9]). Bupropion, listed in international guidelines as an antidepressant specifically recommended for bipolar depression, is administered very rarely. Moreover, antidepressants with a high potential for pharmacokinetic interactions, i.e. paroxetine, fluoxetine and fluvoxamine, are not used within the usual combinations. Hence, critical drug-drug interactions are avoided despite increasing polypharmacy (cf. [16]). A trend to polypharmacy has already been described in the treatment of bipolar disorder generally [17-19], a systematic description of this trend for the treatment of bipolar depression is - to our knowledge - given for the first time in our previous [9] and present analysis.

Within the time period of 2006 – 2009, the anticonvulsants valproic acid and lamotrigine were the third and fourth most frequently given substances for bipolar depression and more than every fifth patient receives valproic acid or lamotrigine in combination with other drugs. The efficacy of valproic acid has been validated in clinical studies and recent meta-analyses [20], whereby the efficacy of lamotrigine is still a controversial issue and only a modest antidepressant effect can be expected [21]. In 2004, an international consensus group on Bipolar I Depression Treatment Guidelines recommended lamotrigine with category 1 evidence [22].

Quetiapine was found in the present study to be the second most frequently prescribed substance for bipolar depression during the period from 2006 to 2009, whereby the number of prescriptions has increased rapidly in the last ten years [9]. In 2010, quetiapine was even the most often prescribed single substance, followed by valproic acid and lithium. Evidence for the efficacy of quetiapine in the treatment of bipolar depression is ample [13,23-26]. Therefore, all international guidelines explicitly recommend quetiapine as first line treatment, usually proposed as monotherapy [1-6]. Already in 2005, in the Texas algorithms for treatment of bipolar I depression quetiapine was proposed besides lamotrigine and olanzapine / fluoxetine combination [27] and in 2007, quetiapine was recommended as first line treatment option by the international CANMAT guidelines [28]. However, monotherapy of quetiapine is very unusual in our data and quetiapine was found to be combined frequently with SSRI and mood stabilizers (lithium, valproic acid or lamotrigine). Note that aripiprazole was prescribed in 2010 in 10% of the patients despite negative trials for bipolar depression [6].

The present study bears some limitations, a detailed description of which is given in Greil et al. 2012 [9]. Most importantly, the study is based on data from hospitalized patients, which suffer from severe depression usually and may be treatment resistant as well. Thus, this population may need a higher number of psychotropic drugs concomitantly as compared to out-patients. Moreover, polypharmacy in our population may be overestimated due to tapering off ineffective drugs and starting new medications.

Overall, the study shows, that multiple combinations of psychotropic substances for therapy of bipolar depression are daily clinical routine. Combinations of antidepressants (SSRI, mirtazapine, venlafaxine) with mood stabilizers (lithium, valproic acid, lamotrigine) and/or atypical neuroleptics (quetiapine, olanzapine, risperidone) are common and combinations of more than one antidepressant substance occur quite often.

The efficacy of these frequent combinations applied has not yet been investigated thoroughly. There is a general agreement, that mood stabilizers (lithium, valpoic acid, lamotrigine) combined with atypical neuroleptics may be efficacious in acute bipolar depression [29-32]. In contrast, the efficacy of combinations with antidepressants, especially with mirtazapine and venlafaxine and the efficacy of combinations involving multiple antidepressant substances are not supported by research data. No studies on the efficacy of mirtazapine for bipolar disorder exist. Concerning venlafaxine, its efficacy in the treatment of bipolar II depression has been shown in small sample sizes only [33-35] and one study shows that venlafaxine may trigger switches to mania [36]. Recent reviews and studies on the efficacy of antidepressants alone or in combination in the treatment of bipolar depression do not find stable effects for their efficacy [37-42]. Indicating awareness of drug safety, combinations with substances that have high interaction properties such as paroxetine, fluoxetine and fluvoxamine were found to be scarce in our data.

## Conclusion

The present study shows that administration of combinations of psychotropic drugs is an every day phenomenon in clinical routine, although profound knowledge about their efficacy is missing. Corresponding recommendations in international guidelines based on clinical trials on the treatment of bipolar depression would constitute great assistance for physicians, but they are not yet available.

## Competing interests

The authors declare that they have no competing interests.

The AMSP Drug Safety Program is organized by the non-profit associations, German, Austrian and Swiss Society of Drug Safety in Psychiatry. Almost all pharmaceutical companies involved in CNS research contribute financial support to the three associations, but they have no influence on the publication.

Educational and research grants since 1993:

Austrian Companies: AstraZeneca Österreich GmbH, Boehringer Ingelheim Austria, Bristol Myers Squibb GmbH, CSC Pharmaceuticals GmbH, Eli Lilly GmbH, Germania Pharma GmbH, GlaxoSmithKline Pharma GmbH, Janssen-Cilag Pharma GmbH, Lundbeck GmbH, Novartis Pharma GmbH, Pfizer Med Inform, Wyeth Lederle Pharma GmbH.

German Companies: Abbott GmbH & Co. KG, AstraZeneca GmbH, Aventis Pharma Deutschland GmbH, Bayer Vital GmbH & Co. KG, Boehringer Mannheim GmbH, Bristol-Myers-Squibb, Ciba Geigy GmbH, Desitin Arzneimittel GmbH, Duphar Pharma GmbH & Co. KG, Eisai GmbH, esparma GmbH Arzneimittel, GlaxoSmithKline Pharma GmbH & Co. KG, Hoffmann-La Roche AG Medical Affairs, Janssen-Cilag GmbH, Janssen Research Foundation, Knoll Deutschland GmbH, Lilly Deutschland GmbH Niederlassung Bad Homburg, Lundbeck GmbH & Co. KG, Novartis Pharma GmbH, Nordmark Arzneimittel GmbH, Organon GmbH, Otsuka-Pharma Frankfurt, Pfizer GmbH, Pharmacia & Upjohn GmbH, Promonta Lundbeck Arzneimittel, Rhone-Poulenc Rohrer Gmbh, Sanofi-Synthelabo GmbH, Sanofi-Aventis Deutschland, Schering AG, SmithKline Beecham Pharma GmbH, Solvay Arzneimittel GmbH, Synthelabo, Arzneimittel GmbH, Dr. Wilmar Schwabe GmbH & Co., Thiemann Arzneimittel GmbH, Troponwerke GmbH & Co. KG, Upjohn GmbH, Wander Pharma GmbH, Wyeth-Pharma GmbH.

Swiss Companies: AHP (Schweiz) AG, AstraZeneca AG, Bristol-Myers, Squibb AG, Desitin Pharma GmbH, Eli Lilly (Suisse) S.A., Essex Chemie AG, GlaxoSmithKline AG, Janssen-Cilag AG, Lundbeck (Suisse) AG, Mepha Pharma AG, Organon AG, Pfizer AG, Pharmacia, Sanofi-Aventis (Suisse) S.A., Sanofi-Synthélabo SA, Servier SA, SmithKlineBeecham AG, Solvay Pharma AG, Wyeth AHP (Suisse) AG, Wyeth Pharmaceuticals AG.

## Authors’ contributions

AH drew the data out of the AMSP data pool, designed and performed the statistical analysis and was drafting the various versions of the manuscript. WG initiated the study, was planning the conception and revised the final version of the manuscript. SR performed another analysis of a corresponding data set thereby validating the results and provided advice for the final version of the manuscript. RG is the project coordinator of AMSP and was counselling the interpretation of the data. All authors read and approved the final manuscript.

## Pre-publication history

The pre-publication history for this paper can be accessed here:

http://www.biomedcentral.com/1471-244X/12/153/prepub
